# From Valve Disease to Valve Replacement: Lipoprotein(a) Is Associated with Aortic Stenosis Severity but Not Prognosis After TAVI

**DOI:** 10.3390/medsci14040484

**Published:** 2026-08-14

**Authors:** Nikolaus Clodi, Nikolaos Schörghofer, Christoph Knapitsch, Bernhard Scharinger, Maximilian Gressl, Gretha Hecke, Michael Lichtenauer, Moritz Brandt, Iris Kremser, Christian Reiter, Hermann Blessberger, Jürgen Kammler, Janne Cadamuro, Clemens Steinwender, Klaus Hergan, Uta C. Hoppe, Martin Clodi, Vera Paar, Elke Boxhammer

**Affiliations:** 1 Department of Radiology, Paracelsus Medical University of Salzburg, Müllner Hauptstraße 48, AT-5020 Salzburg, Austria; n.schoerghofer@salk.at (N.S.); e.boxhammer@salk.at (E.B.); 2 Department of Radiology, Krankenhaus der Barmherzigen Brüder Salzburg, AT-5010 Salzburg, Austria; 3 Department of Internal Medicine II, Division of Cardiology and Intensive Care Medicine, Paracelsus Medical University of Salzburg, AT-5020 Salzburg, Austria; 4 Department of Pharmacy, Paracelsus Medical University of Salzburg, AT-5020 Salzburg, Austria; 5 Department of Medical and Chemical Laboratory Diagnostics, Paracelsus Medical University of Salzburg, AT-5020 Salzburg, Austria; 6 Department of Internal Medicine I, Division of Cardiology and Intensive Care Medicine, Kepler University Hospital, AT-4020 Linz, Austria; 7 Institute for Cardiovascular and Metabolic Research JKU (ICMR), Johannes Kepler University Linz, AT-4020 Linz, Austria; 8 Department of Internal Medicine, Krankenhaus der Barmherzigen Brüder Linz, AT-4021 Linz, Austria

**Keywords:** lipoprotein(a), aortic stenosis, transcatheter aortic valve implantation, aortic valve calcification, prognosis, valve hemodynamics

## Abstract

**Background**: Lipoprotein(a) [Lp(a)] has emerged as a key mediator of calcific aortic valve disease and is strongly linked to the development and progression of aortic stenosis (AS). Whether elevated Lp(a) influences disease severity, long-term prognosis, or both in patients undergoing transcatheter aortic valve implantation (TAVI) remains uncertain. **Methods**: This multicenter study included 213 patients with severe symptomatic AS undergoing TAVI between 2016 and 2018. Pre-procedural Lp(a) concentrations were measured using a standardized immunoturbidimetric assay. Associations with long-term all-cause mortality were evaluated using Kaplan–Meier analyses and Cox proportional hazards models. The relationship between Lp(a) and AS severity was assessed using correlation analyses, multivariable linear regression models, and restricted cubic spline analyses. Anatomical and hemodynamic measures included aortic valve calcium score (AVCS), peak aortic jet velocity (AV Vmax), and transvalvular pressure gradients. **Results**: During a median follow-up of 7.58 years (IQR 4.33–8.66 years), Lp(a) was not associated with all-cause mortality, irrespective of whether it was analyzed as a continuous variable, according to established clinical thresholds, or across quartiles. In contrast, higher Lp(a) concentrations were associated with greater valvular calcification and more severe hemodynamic obstruction. These associations remained significant after adjustment for age, sex, diabetes mellitus, and renal function. Restricted cubic spline analyses suggested predominantly linear associations without evidence of significant non-linearity. **Conclusions**: Elevated Lp(a) concentrations were associated with greater anatomical and hemodynamic severity of AS but not with long-term mortality after TAVI. These findings suggest that elevated Lp(a) is associated with greater anatomical and hemodynamic severity of calcific aortic valve disease but was not linked to long-term mortality after TAVI.

## 1. Introduction

Calcific aortic stenosis (AS) is the most common valvular heart disease in developed countries and represents a major cause of morbidity and mortality among elderly individuals. With increasing life expectancy and expanding indications for transcatheter aortic valve implantation (TAVI), the number of patients undergoing treatment for severe AS continues to rise. Although degenerative AS was historically considered a passive consequence of aging, increasing evidence suggests that the disease is an active and highly regulated process involving lipid deposition, chronic inflammation, and progressive valvular calcification [1,2,3].

Among the factors implicated in the pathogenesis of calcific AS, lipoprotein(a) [Lp(a)] has emerged as one of the most important genetically determined cardiovascular risk factors. Elevated Lp(a) concentrations promote the transport of oxidized phospholipids into the aortic valve, thereby stimulating inflammatory signaling pathways and osteogenic transformation of valvular interstitial cells, ultimately accelerating valve calcification [4,5,6]. Large epidemiological, genetic, and imaging studies have consistently demonstrated associations between elevated Lp(a) concentrations and the presence, progression, and calcific burden of aortic valve disease [7,8,9].

Despite the established role of Lp(a) in the development of AS, its clinical relevance after valve replacement remains less well defined. Once severe symptomatic AS has developed and the diseased valve is replaced, the mechanisms driving long-term prognosis may differ substantially from those involved in disease initiation and progression. In elderly TAVI populations, survival is influenced by a broad spectrum of factors, including age, frailty, comorbidities, renal dysfunction, and concomitant cardiovascular disease [10,11,12,13]. Consequently, it remains uncertain whether Lp(a) continues to exert prognostic effects after successful valve intervention or primarily reflects the severity of the underlying valvular disease.

Therefore, the aim of the present study was to evaluate the prognostic significance of pre-procedural Lp(a) concentrations for long-term all-cause mortality in patients undergoing TAVI. In addition, we investigated the association between Lp(a) and anatomical as well as hemodynamic markers of AS severity, including aortic valve calcium score (AVCS), peak aortic jet velocity (AV Vmax), and transvalvular pressure gradients.

## 2. Materials and Methods

### 2.1. Study Population

This retrospective multi-center study assessed 260 consecutive patients with severe symptomatic AS who underwent TAVI at two tertiary referral centers in Austria (Paracelsus Medical University Salzburg and Kepler University Hospital Linz) between January 2016 and April 2018. All patients were evaluated by a multidisciplinary heart team and underwent comprehensive pre-procedural clinical, laboratory, echocardiographic, and computed tomography assessments according to institutional standards and contemporary guideline recommendations.

**Figure 1:**
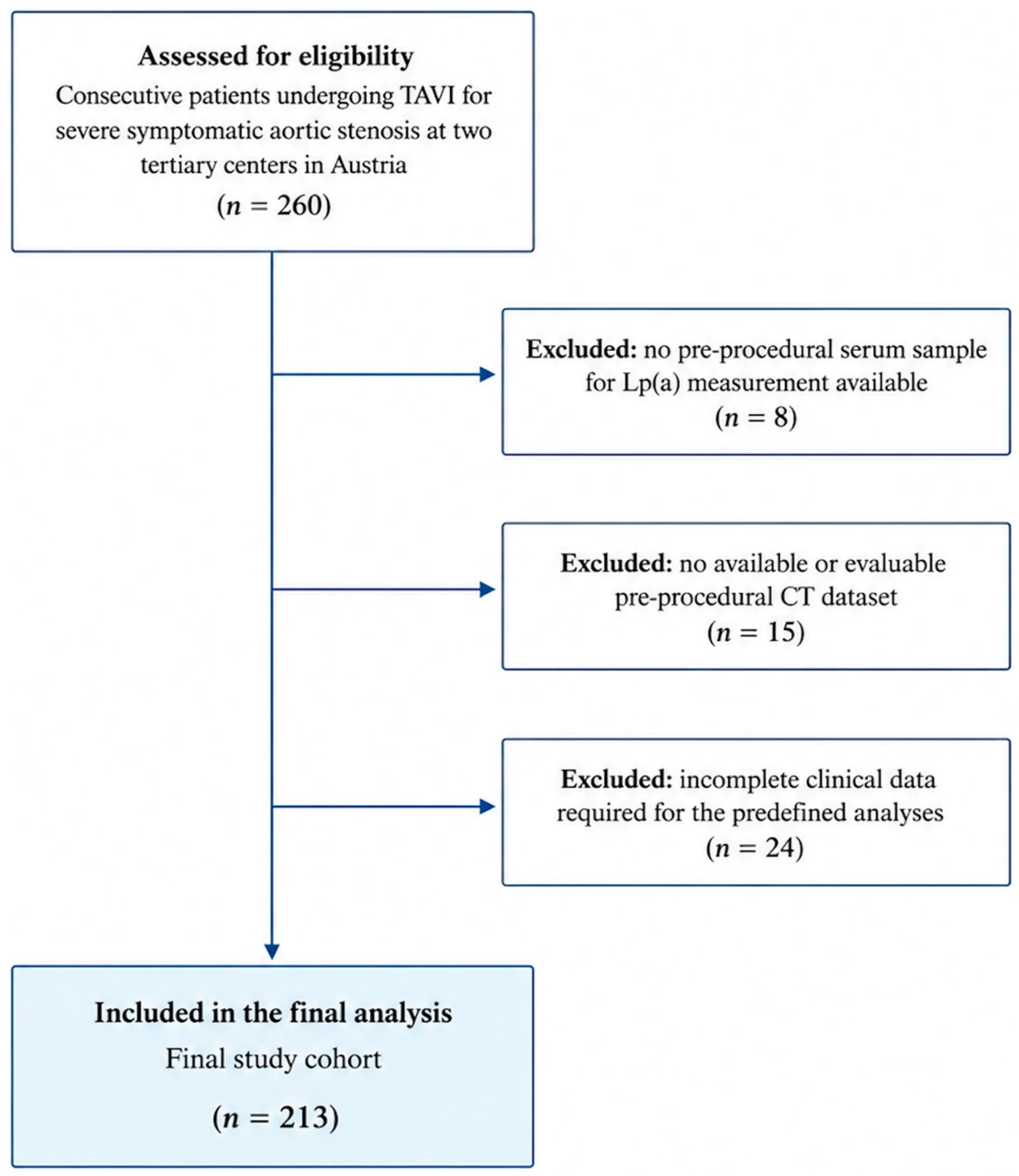
STROBE flow diagram of study cohort selection. Among 260 consecutive patients undergoing TAVI at two tertiary referral centers between January 2016 and April 2018, 8 were excluded because no pre-procedural serum sample was available for Lp(a) measurement, 15 were excluded because no available or evaluable pre-procedural computed tomography dataset for aortic valve calcium scoring was available and 24 incomplete clinical datasets led to exclusion. The final study cohort comprised 213 patients included in the analyses of long-term all-cause mortality and aortic stenosis severity.

Patients were eligible for the present analysis if a pre-procedural serum sample for Lp(a) measurement, an available and evaluable pre-procedural computed tomography dataset for AVCS, and complete clinical data required for the predefined analyses were available. Of the 260 patients assessed for eligibility, 8 were excluded because no pre-procedural serum sample was available, 15 because no available or evaluable pre-procedural CT dataset was available, and 24 because of incomplete clinical data required for the predefined analyses. The final study cohort therefore comprised 213 patients, as illustrated in the STROBE diagram in Figure 1.

Missing data were handled using a complete-case approach. Patients with missing laboratory, imaging, or clinical variables required for the predefined analyses were excluded before statistical analysis. Consequently, all primary correlation, regression, and restricted cubic spline analyses were performed in the final study cohort (*n* = 213), and no imputation of missing values was performed.

Baseline clinical characteristics, laboratory parameters, imaging findings, procedural data, and follow-up information were retrospectively obtained from institutional electronic medical records and archived databases.

### 2.2. Ethical Considerations

The study was conducted in accordance with the principles of the Declaration of Helsinki and Good Clinical Practice. The study protocol was approved by the local ethics committees of Paracelsus Medical University Salzburg, Austria (415-E/1969/5–2016, date: 25 January 2016), and Johannes Kepler University Linz, Austria (E-41–16, date: 1 April 2016). Due to the retrospective study design and the use of anonymized routinely collected clinical data, the requirement for written informed consent was waived by the respective ethics committees.

### 2.3. Laboratory Assessment of Lp(a)

Venous blood samples were obtained during routine pre-procedural evaluation prior to TAVI. Serum samples were processed according to institutional protocols and stored at −70 °C until analysis.

All Lp(a) measurements were performed in a single central laboratory at Paracelsus Medical University Salzburg using the particle-enhanced immunoturbidimetric Tina-quant^®^ Lipoprotein(a) Gen.2 assay (Roche Diagnostics International Ltd., Rotkreuz, Switzerland) on a cobas^®^ clinical chemistry analyzer (Roche Diagnostics, Mannheim, Germany). The assay is standardized against an apo(a) isoform-independent reference method and demonstrates excellent analytical precision according to the manufacturer’s specifications. Results were reported in mg/dL according to the manufacturer’s specifications.

For statistical analyses, Lp(a) was evaluated as a continuous variable and after logarithmic transformation. In addition, patients were categorized according to established clinical thresholds of ≥30 mg/dL and ≥50 mg/dL as well as sex-independent quartiles of the study population.

### 2.4. Transthoracic Echocardiography

Comprehensive transthoracic echocardiography (TTE) was performed routinely within 1–4 weeks prior to TAVI using commercially available ultrasound systems (iE33 or EPIQ 5, Philips Healthcare, Hamburg, Germany). All examinations were conducted by experienced cardiologists with advanced training in echocardiography.

Severe AS was diagnosed according to contemporary European Society of Cardiology guideline recommendations. Left ventricular ejection fraction (LVEF) was assessed using the Simpson biplane method. Interventricular septal thickness at end-diastole (IVSd) was measured from standard parasternal long-axis views.

Markers of AS severity, including AV Vmax, peak transvalvular pressure gradient (AVMAX), and mean transvalvular pressure gradient (AVMPG), were obtained using continuous-wave Doppler interrogation of the aortic valve and recorded from routine clinical examinations. Effective orifice area was not included in the present analyses because its calculation relies on the continuity equation and is highly dependent on accurate measurement of the left ventricular outflow tract (LVOT) diameter. As small inaccuracies in LVOT diameter measurements may substantially influence the calculated valve area, particularly in retrospective multicenter datasets, directly measured Doppler-derived parameters were selected as the primary hemodynamic markers of aortic stenosis severity. These echocardiographic parameters were used for correlation analyses and multivariable regression models investigating the association between LP(a) and hemodynamic AS severity.

### 2.5. Computed Tomography and AVCS

As part of the routine pre-procedural assessment for TAVI, all patients underwent ECG gated non-contrast cardiac computed tomography and contrast-enhanced computed tomography angiography. Image acquisition was performed using multidetector CT scanners (Revolution, General Electric Healthcare, Chicago, IL, USA, or Somatom Definition AS+, Siemens Healthcare, Erlangen, Germany) according to institutional imaging protocols.

AVCS was performed on non-contrast CT datasets by an experienced radiologist specialized in cardiovascular imaging using dedicated post-processing software (Dedalus HealthCare, DeepUnity Diagnost, version 2.4.0.1, Bonn, Germany, and IntelliSpace, Philips Healthcare, Advanced Visualization Workspace, version 15.0, Amsterdam, The Netherlands). Calcified lesions of the aortic valve leaflets were identified and quantified according to the Agatston method. Briefly, areas with an attenuation of ≥130 Hounsfield Units (HU) were automatically detected, and lesion-specific scores were calculated by multiplying the calcified area by a density weighting factor based on peak attenuation. Individual lesion scores were summed to obtain the total AVCS, which was expressed in Agatston Units (AU).

Aortic valve calcium quantification was performed in accordance with established Society of Cardiovascular Computed Tomography recommendations and previously published institutional protocols. AVCS served as the primary anatomical marker of AS severity and was analyzed in relation to pre-procedural LP(a) concentrations.

### 2.6. Follow-Up and Clinical Outcomes

The primary clinical endpoint of the study was long-term all-cause mortality following transcatheter aortic valve implantation (TAVI). Follow-up time was calculated from the date of TAVI until death or the last available clinical follow-up. Survival status was determined using institutional electronic medical records, outpatient documentation, correspondence with referring physicians, and official mortality records when available.

### 2.7. Statistical Analysis

Statistical analyses were performed using R version 4.2.3 (R Foundation for Statistical Computing, Vienna, Austria).

Continuous variables are presented as median and interquartile range (IQR), whereas categorical variables are reported as absolute frequencies and percentages. Comparisons between groups were performed using the Mann–Whitney U test for continuous variables and the chi-square test or Fisher’s exact test for categorical variables, as appropriate.

Long-term survival following TAVI was assessed using Kaplan–Meier methodology and compared by log-rank testing. Survival analyses were performed according to clinically established Lp(a) thresholds of 30 mg/dL and 50 mg/dL as well as according to quartiles of the Lp(a) distribution. Cox proportional hazards regression models were used to investigate the association between pre-procedural Lp(a) concentrations and all-cause mortality. Lp(a) was analyzed as a continuous variable after logarithmic transformation and according to predefined threshold categories. Multivariable Cox regression models were adjusted for age, sex, diabetes mellitus, coronary artery disease, atrial fibrillation (AF), and estimated glomerular filtration rate (eGFR). Hazard ratios (HRs) are reported with corresponding 95% confidence intervals (CIs). With 127 deaths and seven parameters included in the fully adjusted Cox model, the analysis provided approximately 18 events per model parameter. The proportional-hazards assumption was assessed using Schoenfeld residuals. Potential center effects were evaluated in sensitivity analyses by additionally adjusting the fully adjusted Cox model for study center and by fitting a Cox model stratified by center.

Lp(a) concentrations and markers of AS severity were further explored using Spearman rank correlation analysis. To assess the association of Lp(a) with anatomical and hemodynamic measures of AS severity, multivariable linear regression models were constructed with AVCS, AV Vmax, AVMPG, and AVMAX as dependent variables. Based on clinical relevance and biological plausibility, all models were adjusted for age, sex, diabetes mellitus, and eGFR. Potential effect modification by sex was assessed by including an Lp(a)-by-sex interaction term in the multivariable linear regression models. To account for potential center-related heterogeneity, sensitivity analyses were additionally performed with the study center included as a covariate. Due to its right-skewed distribution, AVCS was logarithmically transformed before regression analysis.

Model assumptions were evaluated by inspection of residual distributions and assessment of heteroscedasticity, multicollinearity, and influential observations. Multicollinearity was assessed using variance inflation factors (VIFs), and influential observations were evaluated using Cook’s distance. Heteroscedasticity was assessed using the Breusch–Pagan test, with heteroscedasticity-consistent (HC3) standard errors used in sensitivity analyses where appropriate. Sensitivity analyses were additionally performed using log-transformed Lp(a) concentrations. To account for multiple testing across the four severity outcomes, *p*-values of the primary multivariable analyses were adjusted using the Holm method. Effect estimates are additionally presented per 50 mg/dL increase in Lp(a) to facilitate clinical interpretation.

To further characterize potential dose–response relationships and evaluate non-linear associations across the entire range of Lp(a) concentrations, restricted cubic spline regression analyses were performed for AVCS, AV Vmax, AVMPG, and AVMAX using four knots placed at the default Harrell percentiles. The median Lp(a) concentration served as the reference value. Overall and non-linear Wald statistics were calculated to assess the significance and shape of the associations.

Unless otherwise specified, all statistical analyses were performed in the final study cohort (n = 213). All statistical tests were two-sided, and a *p*-value < 0.05 was considered statistically significant.

## 3. Results

### 3.1. Study Cohort

Between January 2016 and April 2018, a total of 213 patients with severe symptomatic AS undergoing TAVI at the participating centers were included in the present analysis. Serum samples had been prospectively collected during routine pre-procedural assessment and stored for subsequent biomarker analyses.

Follow-up information was available for all 213 patients (100%). During a median follow-up of 7.58 years (IQR 4.33–8.66 years), 127 patients (59.6%) died, while 86 patients (40.4%) were censored at their last available follow-up. The maximum follow-up duration was 10.06 years.

The median age of the study population was 84 years (IQR 80–86 years), and 106 patients (49.8%) were female. Median pre-procedural Lp(a) concentration was 10.3 mg/dL (IQR 5.3–25.0 mg/dL).

All patients underwent TAVI using self-expanding transcatheter heart valves. According to the predefined threshold of 50 mg/dL, 39 patients (18.3%) exhibited elevated Lp(a) concentrations, whereas 174 patients (81.7%) had Lp(a) levels below 50 mg/dL.

Baseline demographic, clinical, laboratory, echocardiographic, and computed tomography characteristics are summarized in Table 1.

### 3.2. Baseline Characteristics According to Lp(a) Status

Baseline characteristics according to Lp(a) status are also summarized in Table 1.

Overall, patients with elevated Lp(a) concentrations (≥50 mg/dL) exhibited a clinical profile largely comparable to those with Lp(a) levels < 50 mg/dL. No significant differences were observed regarding cardiovascular risk factors, renal function, or major comorbidities, including diabetes mellitus, arterial hypertension, coronary artery disease, AF, prior myocardial infarction, prior stroke, peripheral artery occlusive disease, and chronic obstructive pulmonary disease.

Similarly, laboratory parameters, including serum creatinine, eGFR, hemoglobin, and NT-proBNP concentrations, did not differ significantly between groups. Left ventricular ejection fraction was comparable irrespective of Lp(a) status.

Patients with elevated Lp(a) concentrations showed numerically higher measures of AS severity, including interventricular septal thickness, AV Vmax, AVMPG, AVMAX, and AVCS. However, these differences did not reach statistical significance in unadjusted analyses.

### 3.3. Association of Lp(a) with Long-Term Mortality

Kaplan–Meier analyses demonstrated no significant association between pre-procedural Lp(a) concentrations and long-term all-cause mortality after TAVI.

**Figure 2:**
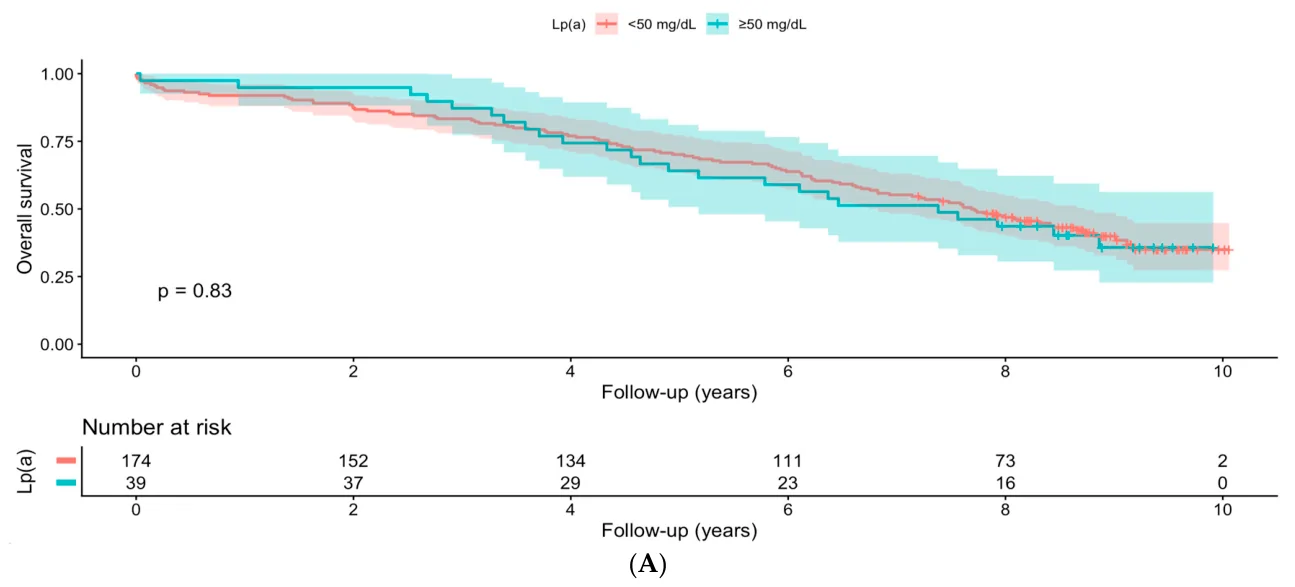

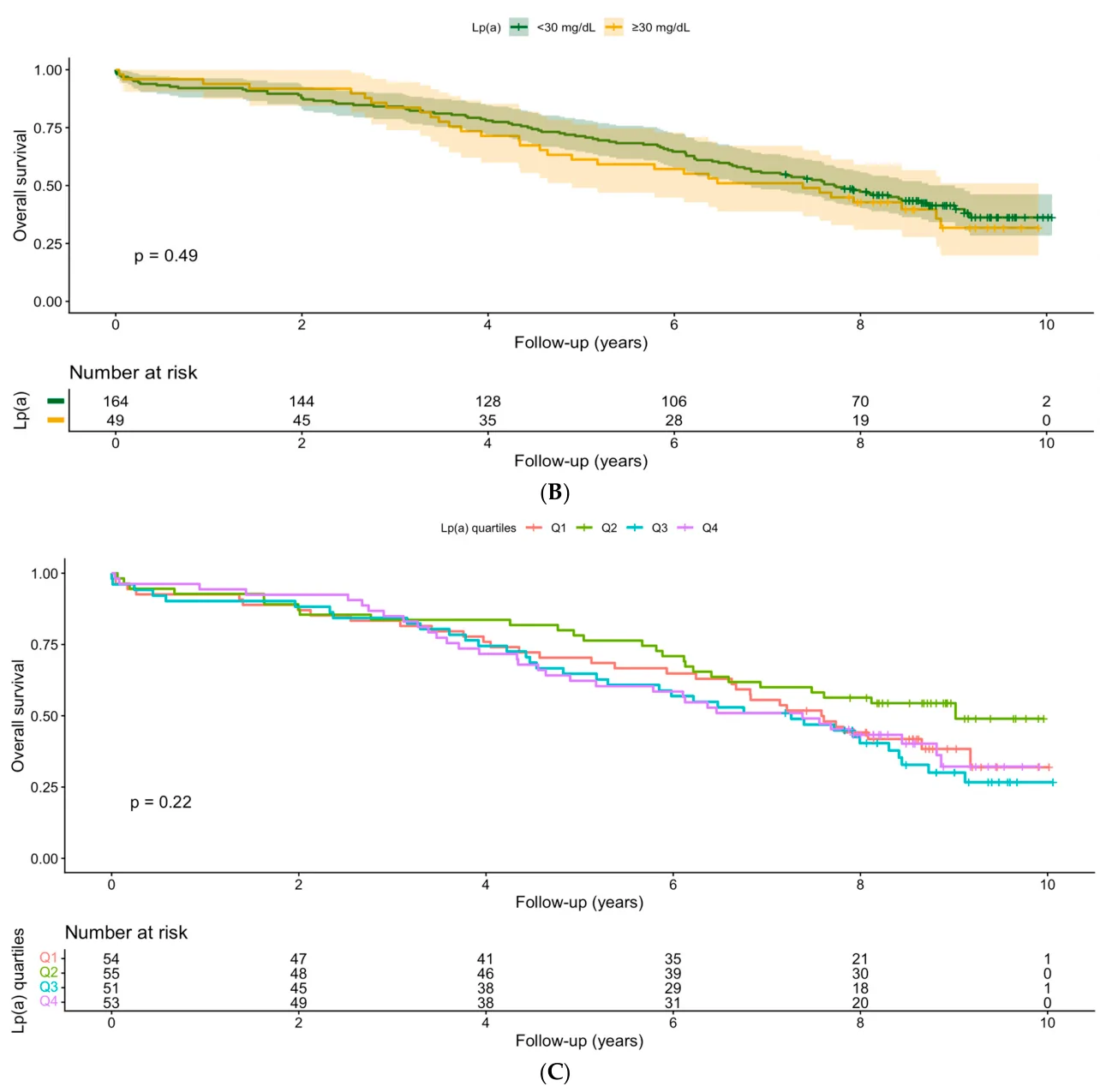
Long-term survival according to LP(a) concentrations. Kaplan–Meier curves illustrating all-cause mortality following TAVI stratified according to established Lp(a) thresholds. (**A**) Lp(a) < 50 mg/dL versus ≥ 50 mg/dL. (**B**) Lp(a) < 30 mg/dL versus ≥ 30 mg/dL. (**C**) Lp(a) quartiles. No significant differences in long-term survival were observed across any Lp(a) categorization. Shaded areas represent 95% CI. Numbers at risk are displayed below the plots. Lp(a) = lipoprotein(a); AVCS = aortic valve calcium score; AV Vmax = peak aortic jet velocity; AVMPG = mean transvalvular pressure gradient; AVMAX = peak transvalvular pressure gradient; Cl = confidence interval.

When stratified according to the clinically established threshold of 50 mg/dL, survival rates were comparable between patients with elevated and non-elevated Lp(a) concentrations throughout follow-up (log-rank *p* = 0.83; Figure 2A). Similar results were observed using a threshold of 30 mg/dL, with no significant differences in long-term survival between groups (log-rank *p* = 0.49; Figure 2B).

To further explore potential threshold-independent effects, patients were additionally categorized according to quartiles of the Lp(a) distribution. Again, Kaplan–Meier analysis revealed no significant differences in long-term mortality among quartiles (log-rank *p* = 0.22; Figure 2C).

### 3.4. Cox Regression Analysis of Long-Term Mortality

To evaluate the prognostic impact of pre-procedural Lp(a) concentrations after TAVI, Cox proportional hazards regression analyses were performed using multiple modeling approaches (Table 2).

When analyzed as a continuous variable, Lp(a) was not associated with long-term all-cause mortality (HR 1.001, 95% CI 0.997–1.005, *p* = 0.688). Similar results were observed after logarithmic transformation of Lp(a) values (HR 1.063, 95% CI 0.918–1.229, *p* = 0.413).

Likewise, categorization according to clinically established thresholds showed no prognostic significance. Patients with Lp(a) concentrations ≥30 mg/dL did not exhibit an increased mortality risk compared with those below this threshold (HR 1.152, 95% CI 0.769–1.728, *p* = 0.492). Similar findings were observed for the threshold of 50 mg/dL (HR 1.049, 95% CI 0.673–1.636, *p* = 0.833).

Adjustment for age and sex did not materially alter the results (HR 1.001, 95% CI 0.997–1.005, *p* = 0.700). Furthermore, in a fully adjusted model including age, sex, diabetes mellitus, coronary artery disease, AF, and eGFR, Lp(a) remained unassociated with long-term mortality (HR 1.000, 95% CI 0.996–1.004, *p* = 0.931).

The proportional-hazards assumption was assessed using Schoenfeld residuals and showed no evidence of a relevant violation for Lp(a) or for the model globally. In sensitivity analyses accounting for the study center, the association between Lp(a) and long-term mortality remained unchanged. Additional adjustment for study center yielded an HR of 1.001 (95% CI 0.997–1.004; *p* = 0.768) for Lp(a), while a Cox model stratified by study center provided comparable results (HR 1.001, 95% CI 0.997–1.004; *p* = 0.764).

Overall, pre-procedural Lp(a) concentrations were not associated with long-term survival following TAVI, irrespective of the analytical approach or degree of multivariable adjustment.

### 3.5. Correlation of Lp(a) with Markers of AS Severity

Spearman rank correlation analyses demonstrated only weak positive correlations between pre-procedural Lp(a) concentrations and markers of AS severity (Table 3).

Lp(a) showed a weak correlation with AVCS (Spearman’s rho = 0.131, *p* = 0.125), AV Vmax (rho = 0.138, *p* = 0.064), AVMPG (rho = 0.120, *p* = 0.088), and AVMAX (rho = 0.121, *p* = 0.087).

Although all correlation coefficients were positive, none reached conventional statistical significance. Although all correlation coefficients were positive, none reached conventional statistical significance, indicating only weak unadjusted associations between Lp(a) and the evaluated markers of AS severity.

### 3.6. Multivariable Association of Lp(a) with Anatomical and Hemodynamic AS Severity

Multivariable linear regression analyses adjusted for age, sex, diabetes mellitus, and eGFR were performed to evaluate the association between Lp(a) and markers of AS severity (Table 4 and Supplementary Tables S1–S4).

Higher Lp(a) concentrations were associated with greater anatomical and hemodynamic AS severity. Per 50 mg/dL higher Lp(a), log-transformed AVCS increased by 0.097 (95% CI 0.013–0.182; *p* = 0.024), corresponding to approximately 10.2% higher AVCS. Corresponding increases were 0.120 m/s for AV Vmax (95% CI 0.035–0.206; *p* = 0.006), 3.07 mmHg for AVMPG (95% CI 1.16–4.99; *p* = 0.0018), and 4.50 mmHg for AVMAX (95% CI 1.49–7.50; *p* = 0.0036). All four associations remained significant after Holm correction for multiple testing (Table 4).

Model diagnostics showed no relevant multicollinearity (all VIFs ≤ 1.15), and residual distributions were generally unremarkable. Assessment of influential observations using Cook’s distance did not indicate that the principal findings were driven by individual observations. Evidence of heteroscedasticity was observed for the AVMPG model; however, the association with Lp(a) remained significant when HC3 robust standard errors were applied (*p* = 0.030). The adjusted R^2^ values were 0.241 for log(AVCS), 0.027 for AV Vmax, 0.032 for AVMPG, and 0.027 for AVMAX. The overall models were significant for log(AVCS) (*p* < 0.001) and AVMPG (*p* = 0.045), but not for AV Vmax (*p* = 0.084) or AVMAX (*p* = 0.066), indicating limited explanatory power of the latter models. Sensitivity analyses using log-transformed Lp(a) showed directionally consistent associations, which remained significant for AV Vmax (*p* = 0.031), AVMPG (*p* = 0.006), and AVMAX (*p* = 0.028), but not for log(AVCS) (*p* = 0.065).

There was no evidence of effect modification by sex for the association between Lp(a) and log-transformed AVCS (*p* for interaction = 0.557), AV Vmax (*p* = 0.773), AVMPG (*p* = 0.921), or AVMAX (*p* = 0.806). In sensitivity analyses additionally adjusted for study center, the associations between Lp(a) and all four measures of AS severity remained materially unchanged.

### 3.7. Dose–Response Relationship Between Lp(a) and AS Severity

**Figure 3:**
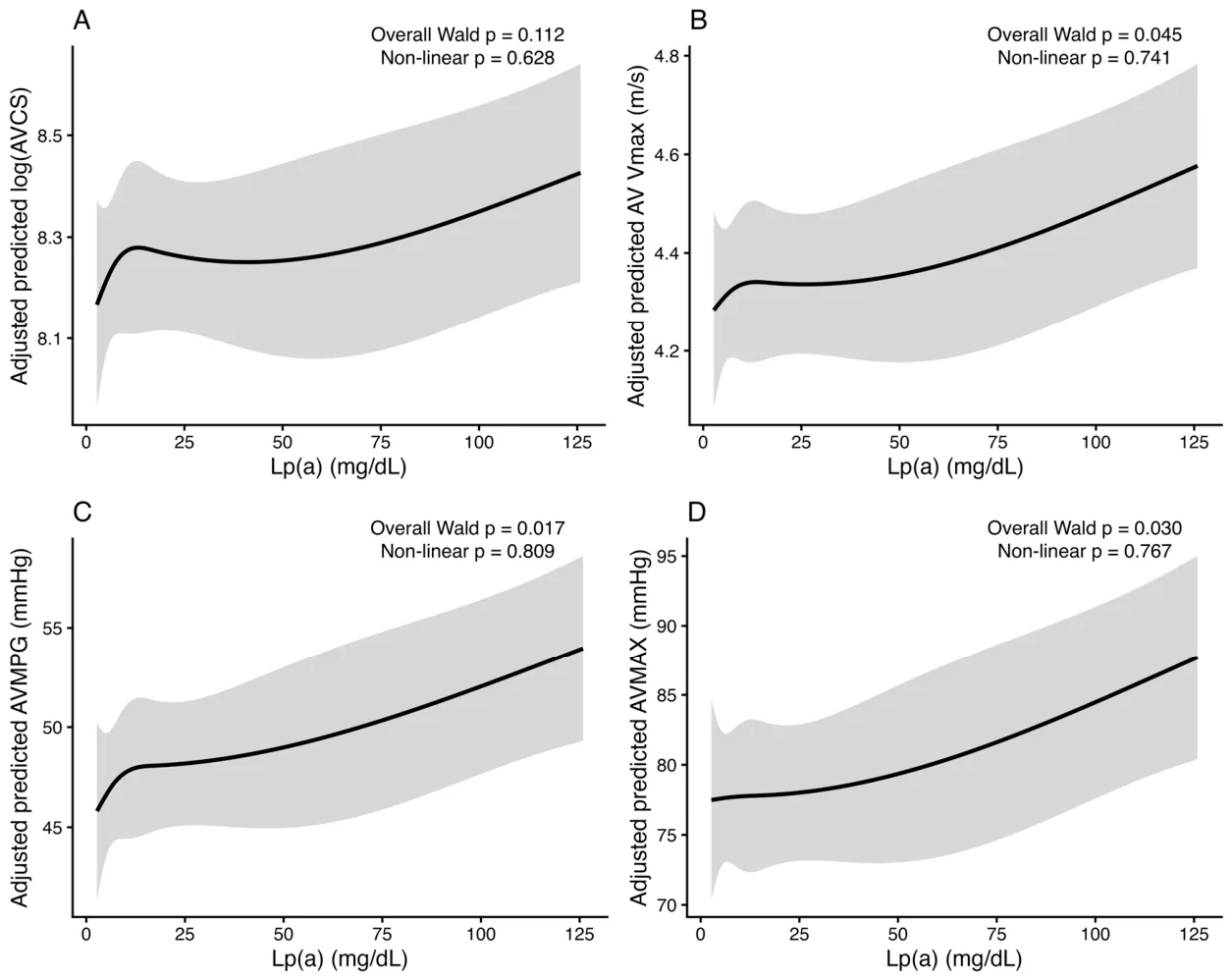
Restricted cubic spline analyses of the association between Lp(a) and markers of aortic stenosis severity. Restricted cubic spline models demonstrating the adjusted relationship between Lp(a) concentrations and (**A**) log-transformed aortic valve calcium score (AVCS), (**B**) peak aortic jet velocity (AV Vmax), (**C**) mean transvalvular pressure gradient (AVMPG), and (**D**) peak transvalvular pressure gradient (AVMAX). Solid lines represent adjusted predicted values, while shaded areas indicate 95% CI. Overall and non-linear Wald *p*-values are shown for each model. The analyses suggest predominantly linear associations between increasing Lp(a) concentrations and markers of aortic stenosis severity. TAVI = transcatheter aortic valve implantation; Lp(a) = lipoprotein(a); AV = aortic valve; Cl = confidence interval.

Restricted cubic spline analyses were performed to further characterize the relationship between Lp(a) concentrations and markers of AS severity across the entire range of observed values (Figure 3).

For all evaluated parameters, spline models suggested a generally positive dose–response relationship between increasing Lp(a) concentrations and greater anatomical or hemodynamic stenosis severity. Significant overall associations were observed for AV Vmax, AVMPG, and AVMAX, whereas a similar positive trend was observed for AVCS.

Importantly, there was no evidence of significant non-linear associations for any of the evaluated endpoints. CI widened at higher Lp(a) concentrations because relatively few patients exhibited markedly elevated Lp(a) levels. Therefore, the spline analyses support predominantly linear associations within the observed data but should not be interpreted as proving a definitive linear dose–response relationship across the entire Lp(a) concentration range.

## 4. Discussion

### 4.1. Principal Findings

The present study demonstrates differing associations of Lp(a) with AS severity and long-term outcome in patients undergoing TAVI. Higher Lp(a) concentrations were associated with greater aortic valve calcification and more severe hemodynamic obstruction after multivariable adjustment, whereas no association with long-term all-cause mortality was detected during extended follow-up.

This apparent dissociation between markers of disease severity and post-procedural outcome represents the central finding of the present study. However, given the retrospective design and the absence of longitudinal assessment of AS progression or a non-TAVI comparator group, these findings should be interpreted as associations and do not establish differential biological effects of Lp(a) before and after valve replacement.

### 4.2. Lp(a) and Long-Term Mortality After TAVI

A central finding of the present study is the absence of an association between pre-procedural Lp(a) concentrations and long-term mortality following TAVI. This observation was remarkably consistent across all analytical approaches, including established clinical thresholds, quartile-based analyses, and multivariable-adjusted Cox regression models.

From a clinical perspective, the absence of an association between Lp(a) and long-term mortality after TAVI may reflect the fundamental difference between disease development and disease outcome. Elevated Lp(a) is well established as a risk factor for the development of calcific AS [4,8,14]. However, once severe symptomatic disease has been diagnosed and successfully treated by valve replacement, the determinants of subsequent survival are likely to change substantially.

Patients undergoing TAVI are typically elderly and characterized by a high burden of cardiovascular and non-cardiovascular comorbidities [10,15]. In this setting, long-term outcome is influenced by a broad spectrum of competing risk factors including frailty [16], renal dysfunction [12,17], heart failure [18], AF [13,19], pulmonary disease [20,21], and overall biological age. Consequently, the relative contribution of a single disease-promoting factor such as Lp(a) may become increasingly diluted during follow-up.

Importantly, TAVI effectively removes the hemodynamic consequences of severe AS, which represent the final common pathway through which progressive valve disease leads to symptoms, ventricular dysfunction, and ultimately death [1,2]. Accordingly, the absence of a detected association with mortality in the present cohort may reflect the predominance of other prognostic factors after TAVI; however, our data cannot determine whether the prognostic relevance of Lp(a) changes as a consequence of valve replacement [22,23].

### 4.3. Lp(a) and AS Severity

Elevated Lp(a) concentrations were associated with both anatomical and hemodynamic markers of AS severity, including AVCS, AV Vmax, and transvalvular pressure gradients.

Interestingly, the observed correlations between Lp(a) and markers of AS severity were weak and did not reach statistical significance in univariable analyses. At first glance, this may appear inconsistent with the significant findings obtained in the adjusted regression models. However, calcific AS is a multifactorial disease influenced by numerous demographic, clinical, and metabolic factors [24]. Age, sex, diabetes mellitus, renal function, and other patient characteristics contribute substantially to interindividual variability in both valve calcification and hemodynamic disease severity [25,26], potentially obscuring the contribution of Lp(a) in simple bivariate analyses.

This interpretation is supported by the multivariable regression models, in which adjustment for major confounders consistently revealed significant associations between Lp(a) and all investigated markers of stenosis severity. Rather than suggesting a dominant effect, these findings indicate that Lp(a) represents one component within a complex network of factors associated with greater anatomical and hemodynamic disease severity. Consistent with these findings, restricted cubic spline analyses did not provide evidence of significant non-linearity, although CI widened at higher Lp(a) concentrations because only a limited number of patients exhibited markedly elevated Lp(a) levels.

### 4.4. Potential Mechanisms Linking Lp(a) to Valvular Calcification

The association between elevated Lp(a) concentrations and greater AS severity observed in our study is biologically plausible. Lp(a) is the major circulating carrier of oxidized phospholipids, which promote inflammatory and pro-calcific processes within the aortic valve and have consistently been linked to the development and progression of calcific AS [5,27,28].

Interestingly, our findings were not limited to aortic valve calcium burden but also extended to hemodynamic markers of disease severity. While calcification represents a structural hallmark of the disease [2], AVMAX, AVMPG and AV Vmax reflect its functional consequences. The observed associations with both structural and hemodynamic measures are consistent with previous experimental and longitudinal studies linking Lp(a) to calcific aortic valve disease.

This observation may be particularly relevant because the severity of calcific AS is not determined solely by calcium burden. Rather, it reflects the combined effects of progressive calcification, leaflet thickening, reduced leaflet mobility, and the resulting deterioration of valve hemodynamics [2,29]. The association of Lp(a) with both anatomical and hemodynamic measures therefore suggests that Lp(a) may be linked to the cumulative pathological processes underlying more advanced calcific aortic valve disease rather than merely reflecting established valve calcification.

### 4.5. Clinical Implications

The present findings suggest that Lp(a) may be more useful as a marker of disease burden than as a prognostic marker in patients undergoing TAVI. While elevated Lp(a) concentrations were associated with greater anatomical and hemodynamic severity of AS, they did not identify patients at increased risk of long-term mortality after valve replacement. These findings highlight the potential relevance of Lp(a) as a marker associated with AS disease burden. Whether Lp(a)-targeted interventions can modify the development or progression of calcific AS, particularly at earlier disease stages, requires evaluation in dedicated prospective interventional studies.

### 4.6. Limitations

Several limitations should be considered when interpreting the present findings. The retrospective study design precludes conclusions regarding causality and introduces the possibility of residual confounding despite multivariable adjustment.

Lp(a) concentrations were determined from stored serum samples at a single pre-procedural time point. Serial measurements before and after TAVI were therefore unavailable, precluding the assessment of temporal changes following valve replacement. However, as Lp(a) concentrations are largely genetically determined and exhibit relatively low intra-individual variability throughout adult life, a single baseline measurement is generally considered representative of an individual’s long-term Lp(a) status.

Although the Lp(a) assay is designed to minimize apo(a) isoform-dependent measurement bias, residual assay-related variability cannot be completely excluded.

The study population consisted exclusively of patients with severe symptomatic AS treated with self-expanding transcatheter heart valves, which may limit the generalizability of the findings to other patient populations and valve platforms.

All-cause mortality was selected as the primary study endpoint. Although this represents a robust and objectively ascertainable outcome in a retrospective cohort with long-term follow-up, it may have diluted potential associations between Lp(a) and cardiovascular-specific events in this elderly patient population. Cardiovascular mortality, repeat valve intervention, heart failure hospitalization, and thromboembolic events were not consistently available and therefore could not be evaluated.

The moderate sample size may have limited the statistical power to detect smaller associations with long-term mortality, particularly at higher Lp(a) concentrations, as only 39 patients had Lp(a) levels ≥ 50 mg/dL. Therefore, the absence of a statistically significant association should not be interpreted as evidence of the absence of a smaller prognostic effect.

## 5. Conclusions

In patients with severe symptomatic AS undergoing TAVI, higher pre-procedural Lp(a) concentrations were associated with greater anatomical and hemodynamic AS severity after multivariable adjustment. In contrast, no statistically significant association between Lp(a) and long-term all-cause mortality was detected during extended follow-up. These findings support an association between Lp(a) and disease burden in advanced calcific AS, while its prognostic significance after TAVI remains uncertain.

## Figures and Tables

**Table 1:** Baseline characteristics according to Lp(a) status. Baseline demographic, clinical, laboratory, echocardiographic, and computed tomography characteristics of the study population stratified according to an Lp(a) threshold of 50 mg/dL. Continuous variables are presented as median [interquartile range], and categorical variables as n (%).

Variable	Overall (n = 213)	Lp(a) < 50 mg/dL (n = 174)	Lp(a) ≥ 50 mg/dL (n = 39)	*p*-Value
No. of patients	213	174	39	
Age, years	84.0 [80.0–86.0]	84.0 [79.0–86.0]	84.0 [80.0–87.0]	0.500
Lp(a), mg/dL	10.3 [5.3–25.0]	7.7 [4.6–15.5]	87.5 [67.5–126.2]	<0.001
Creatinine, mg/dL	1.0 [0.9–1.3]	1.0 [0.9–1.3]	1.0 [0.9–1.3]	0.624
eGFR, mL/min/1.73 m^2^	62.6 [50.0–70.0]	62.0 [50.0–70.0]	63.1 [51.0–71.0]	0.537
Hemoglobin, g/dL	12.8 [11.6–13.9]	12.9 [11.7–14.0]	12.7 [11.4–13.5]	0.313
NT-proBNP, pg/mL	1294 [600–2726]	1393 [632–2739]	943 [514–2224]	0.439
LVEF, %	60.0 [52.0–63.0]	60.0 [52.5–63.8]	60.0 [52.0–61.5]	0.752
IVSd, mm	15.0 [13.0–17.0]	14.0 [13.0–16.8]	15.5 [14.0–18.0]	0.054
AV Vmax, m/s	4.4 [4.0–4.7]	4.3 [4.0–4.7]	4.5 [4.2–4.9]	0.083
AVMPG, mmHg	48.0 [41.0–58.0]	47.0 [41.0–57.0]	49.5 [43.2–60.0]	0.143
AVMAX, mmHg	77.0 [68.0–90.0]	75.0 [68.0–89.0]	82.5 [70.5–96.8]	0.077
AVCS, AU	2922.9 [2094.5–4282.2]	2826.1 [2094.0–4327.0]	3176.5 [2719.6–4088.3]	0.297
Female sex	106 (49.8)	85 (48.9)	21 (53.8)	0.573
Diabetes mellitus	49 (23.0)	38 (21.8)	11 (28.2)	0.393
Arterial hypertension	170 (79.8)	140 (80.5)	30 (76.9)	0.619
Coronary artery disease	150 (70.4)	122 (70.1)	28 (71.8)	0.835
AF	72 (33.8)	61 (35.1)	11 (28.2)	0.414
Prior MI	7 (3.3)	6 (3.4)	1 (2.6)	1.000
Previous cardiac surgery	12 (5.6)	9 (5.2)	3 (7.7)	0.464
Pacemaker before TAVI	17 (8.0)	16 (9.2)	1 (2.6)	0.322
Prior stroke	12 (5.7)	10 (5.8)	2 (5.3)	1.000
PAOD	14 (6.6)	12 (6.9)	2 (5.1)	1.000
COPD	19 (8.9)	13 (7.5)	6 (15.4)	0.126

Abbreviations:: Lp(a) = lipoprotein(a); eGFR = estimated glomerular filtration rate; LVEF = left ventricular ejection fraction; IVSd = interventricular septal thickness in diastole; AF = atrial fibrillation; AU = Agatston Unit; AV Vmax = peak aortic jet velocity; AVMPG = mean transvalvular pressure gradient; AVMAX = peak transvalvular pressure gradient; AVCS = aortic valve calcium score; PAOD = peripheral artery occlusive disease; COPD = chronic obstructive pulmonary disease; TAVI = transcatheter aortic valve implantation; MI = myocardial infarction.

**Table 2:** Association of pre-procedural Lp(a) concentrations with long-term all-cause mortality after TAVI. Cox proportional hazards regression analyses evaluating the association between pre-procedural Lp(a) concentrations and long-term all-cause mortality. Lp(a) was analyzed as a continuous variable, a log-transformed variable, and according to clinically established thresholds. Multivariable models were adjusted for clinically relevant confounders.

Analysis	HR	95% CI	*p*-Value
Lp(a), continuous	1.001	0.997–1.005	0.688
Lp(a), log	1.063	0.918–1.229	0.413
Lp(a) ≥30 mg/dL	1.152	0.769–1.728	0.492
Lp(a) ≥50 mg/dL	1.049	0.673–1.636	0.833
Lp(a), adjusted for age and sex	1.001	0.997–1.005	0.700
Lp(a), fully adjusted *	1.000	0.996–1.004	0.931

* Adjusted for age, sex, diabetes mellitus, coronary artery disease, AF, and eGFR. Abbreviations: HR = hazard ratio; CI = confidence interval; TAVI = transcatheter aortic valve implantation; Lp(a) = lipoprotein(a).

**Table 3:** Correlation between Lp(a) concentrations and imaging markers of aortic stenosis severity. Spearman correlation analyses assessing the relationship between circulating Lp(a) concentrations and anatomical and hemodynamic markers of aortic stenosis severity.

Variable	Spearman’s Rho	*p*-Value
AVCS	0.131	0.125
AV Vmax	0.138	0.064
Mean transvalvular gradient (AVMPG)	0.120	0.088
Peak transvalvular gradient (AVMAX)	0.121	0.087

Abbreviations: Lp(a) = lipoprotein(a); AVCS = aortic valve calcium scoring; AV Vmax = peak aortic jet velocity; AVMPG = mean transvalvular pressure gradient; AVMAX = peak transvalvular pressure gradient.

**Table 4:** Multivariable associations between Lp(a) concentrations and markers of aortic stenosis severity. Multivariable linear regression models evaluating the association between pre-procedural Lp(a) concentrations and anatomical and hemodynamic markers of aortic stenosis severity. Models were adjusted for age, sex, diabetes mellitus, and eGFR. Regression coefficients (β) represent the change in the respective outcome per 50 mg/dL increase in Lp(a). AVCS was log-transformed because of its right-skewed distribution. *p*-values were adjusted for multiple testing across the four severity outcomes using the Holm method.

Outcome	β for Lp(a)	95% CI	*p*-Value	Holm-Adjusted *p*
log(AVCS)	0.097	0.013–0.182	0.0240	0.024
AV Vmax (m/s)	0.120	0.035–0.206	0.0060	0.012
AVMPG (mmHg)	3.074	1.163–4.985	0.0018	0.007
AVMAX (mmHg)	4.495	1.489–7.501	0.0036	0.011

Abbreviations: Lp(a) = lipoprotein(a); AVCS = aortic valve calcium scoring; AV Vmax = peak aortic jet velocity; AVMPG = mean transvalvular pressure gradient; AVMAX = peak transvalvular pressure gradient.

## Data Availability

The data presented in this study are available on request from the corresponding author. Because the ethics committee approval has imposed ethical and privacy restrictions that prohibit public disclosure of the underlying patient data.

## Supplementary Materials

The following supporting information can be downloaded at: https://www.mdpi.com/article/10.3390/medsci14040484/s1, Table S1: Multivariable linear regression analysis of aortic valve calcification (AVCS); Table S2: Multivariable linear regression analysis of AV Vmax; Table S3: Multivariable linear regression analysis of AVMPG; Table S4: Multivariable linear regression analysis of AVMAX.

## Abbreviations

The following abbreviations are used in this manuscript:

AS	Aortic stenosis
AU	Agatston units
AV Vmax	Peak aortic jet velocity
AVCS	Aortic valve calcium score
AVMAX	Peak transvalvular pressure gradient
AVMPG	Mean transvalvular pressure gradient
CI	Confidence interval
CT	Computed tomography
ECG	Electrocardiography
eGFR	Estimated glomerular filtration rate
HR	Hazard ratio
HU	Hounsfield units
ICMR	Institute for Cardiovascular and Metabolic Research
IQR	Interquartile range
IVSd	Interventricular septal thickness at end-diastole
Lp(a)	Lipoprotein(a)
LVEF	Left ventricular ejection fraction
NT-proBNP	N-terminal pro-B-type natriuretic peptide
TAVI	Transcatheter aortic valve implantation
TTE	Transthoracic echocardiography
