# Supplementary material for: From Valve Disease to Valve Replacement: Lipoprotein(a) Is Associated with Aortic Stenosis Severity but Not Prognosis After TAVI
Source: Med Sci (Basel). 2026 Aug 14;14(4):484. doi: 10.3390/medsci14040484 (PMC13515942; doi:10.3390/medsci14040484)
Supplement: Supplementary file 1 [file medsci-14-00484-s001.zip › medsci-4461385-supplementary.pdf]

Supplementary Table S1. Multivariable linear regression analysis of aortic valve calcification (AVCS)

Variable	β coefficient	95% CI	p-value
Lp(a)	0.00195	0.00026 to 0.00363	0.024
Age	0.01123	-0.00548 to 0.02793	0.186
Female sex	-0.51873	-0.68133 to -0.35613	<0.001
Diabetes mellitus	-0.01670	-0.20677 to 0.17338	0.862
eGFR	0.00146	-0.00367 to 0.00660	0.573

Model characteristics

Parameter	Value
Dependent variable	log(AVCS)
Adjusted R^2	0.241
Overall model p-value	<0.001

The dependent variable was log-transformed AVCS [log (AVCS)]. Regression coefficients (β), 95% confidence intervals (CI), and corresponding p-values are presented for each covariate included in the model. Female sex was associated with lower AVCS values, whereas Lp(a) was independently associated with higher AVCS values. Model characteristics, including the adjusted coefficient of determination (adjusted R^2) and overall model significance, are provided below.
Abbreviations: AVCS = aortic valve calcification score; Lp(a) = lipoprotein(a); CI = confidence interval; eGFR = estimated glomerular filtration rate; β = regression coefficient; R^2 = coefficient of determination.

Supplementary Table S2. Multivariable linear regression analysis of AV Vmax

Variable	β coefficient	95% CI	p-value
Lp(a)	0.00240	0.00069–0.00411	0.006
Age	-0.00693	-0.02429–0.01044	0.432
Female sex	0.04029	-0.11934–0.19993	0.619
Diabetes mellitus	-0.06146	-0.25153–0.12860	0.524
eGFR	-0.00357	-0.00852–0.00137	0.156

Model characteristics

Parameter	Value
Dependent variable	AV Vmax
Adjusted R^2	0.027
Overall model p-value	0.084

Regression coefficients (β), 95% confidence intervals (CI), and corresponding p-values are presented for each covariate included in the model. Lipoprotein(a) [Lp(a)] was independently associated with higher AV Vmax values, whereas age, sex, diabetes mellitus, and estimated glomerular filtration rate (eGFR) were not significantly associated with AV Vmax. Model characteristics, including the adjusted coefficient of determination (adjusted R^2) and overall model significance, are provided below.

Abbreviations: AV = aortic valve; Vmax = peak transvalvular velocity; Lp(a) = lipoprotein(a); CI = confidence interval; eGFR = estimated glomerular filtration rate; β = regression coefficient; R^2 = coefficient of determination.

Supplementary Table S3. Multivariable linear regression analysis of AVMPG

Variable	β coefficient	95% CI	p-value
Lp(a)	0.06148	0.02325–0.09970	0.0018
Age	-0.20772	-0.56150–0.14607	0.248
Female sex	0.84468	-2.57357–4.26293	0.627
Diabetes mellitus	0.39449	-3.62049–4.40946	0.847
eGFR	-0.01998	-0.12089–0.08094	0.697

Model characteristics

**Parameter**	**Value**
Dependent variable	AVMPG
Adjusted R^2	0.032
Overall model p-value	0.045

Regression coefficients (β), 95% confidence intervals (CI), and corresponding p-values are presented for each covariate included in the model. Lipoprotein(a) [Lp(a)] was independently associated with higher AVMPG values, whereas age, sex, diabetes mellitus, and estimated glomerular filtration rate (eGFR) were not significantly associated with AVMPG. Model characteristics, including the adjusted coefficient of determination (adjusted R^2) and overall model significance, are provided below.

Abbreviations: AVMPG = aortic valve mean pressure gradient; Lp(a) = lipoprotein(a); CI = confidence interval; eGFR = estimated glomerular filtration rate; β = regression coefficient; R^2 = coefficient of determination.

Supplementary Table S4. Multivariable linear regression analysis of AVMAX

Variable	β coefficient	95% CI	p-value
Lp(a)	0.08990	0.02978–0.15003	0.0036
Age	-0.06464	-0.62977–0.50050	0.822
Female sex	-0.20114	-5.60331–5.20104	0.942
Diabetes mellitus	-1.99108	-8.35289–4.37073	0.538
eGFR	-0.11967	-0.27849–0.03916	0.139

Model characteristics

Parameter	Value
Dependent variable	AVMAX
Adjusted R^2	0.027
Overall model p-value	0.066

Regression coefficients (β), 95% confidence intervals (CI), and corresponding p-values are presented for each covariate included in the model. Lipoprotein(a) [Lp(a)] was independently associated with higher AVMAX values, whereas age, sex, diabetes mellitus, and estimated glomerular filtration rate (eGFR) were not significantly associated with AVMAX. Model characteristics, including the adjusted coefficient of determination (adjusted R^2) and overall model significance, are provided below.

Abbreviations: AVMAX = aortic valve maximum pressure gradient; Lp(a) = lipoprotein(a); CI = confidence interval; eGFR = estimated glomerular filtration rate; β = regression coefficient; R^2 = coefficient of determination.
